# The End of the Accidental Academician

**DOI:** 10.5811/westjem.2019.10.45555

**Published:** 2019-12-09

**Authors:** Christine R. Stehman

**Affiliations:** Indiana University School of Medicine, Department of Emergency Medicine, Indianapolis, Indiana

## INTRODUCTION

Many medical education scholars form their careers through a unique blend of planning and accident. Although these academicians initially worked hard with focus, once they achieve the clinician part of “clinical educator,” it is as if they lack a conceptual framework for the rest of their career. They have become accidental academicians with the educator part succeeding eventually, cobbled together from satisfying mandates and falling into opportunities with little of the meticulous planning that led to being a clinician. Albeit successful, the resulting career may seem disjointed, unplanned, and possibly less fulfilling than it could have been. It is hard to mentor others to achieve success using this model, resulting in more generations of accidental academicians.

Readers may argue that they did not accidentally get where they are today and a plan continues to direct their careers. But, is hindsight making that vague destination less vague? Were changes in direction well thought out or were they the result of attention-catching “squirrels” being chased until their scent was lost, with educators then wandering aimlessly until they found a new trail? Accidental academicians are not bad – I have been one myself; however, I believe that adding intention to the clinical educator career, changing accidental academicians to deliberate professionals, will make the career journey less arduous and more fulfilling.

Trede and McEwen define deliberate professionals as those who reflect on their culture, environment, and situation to understand possibilities and probabilities, and then use that information to make conscious choices for actions and relationships, while being responsible for the consequences of their choices.^1^ Essentially, deliberate professionals know where they are, where they are going, what they need to get there, and that the **path might change**. They use reflection to understand how their characteristics and experiences affect their journey, building individualized opportunities according to their unique characteristics.

Post board certification, the clinical educator travels a long road, filled with slowdowns, detours, and day trips. Accidental academicians often passively go to their future, traveling without any idea of where they are going, how long they’ll be gone or what they’ll do when they get “there.” Deliberate professionals actively create their future by having an individualized map, specific guidance, and a plan for all parts of their journey.

### The Map

A usable, individualized map consists of a starting point, an ending point, and the multiple paths between the two. Using time and introspection to create this roadmap facilitates a more successful and smooth journey. Directions are always relative to a starting point, so education scholars must understand their current position: a combination of their values, interests, and previously acquired knowledge.^2^ Deliberate professionals use this information to choose a direction to travel and plan their route, allowing them to align their career and values.^3^ This alignment leads to happier and more fulfilling experiences.^4^–^6^

Aligning a career path with vague values (ie, I want to help people) is difficult, and such values often falter when challenged.^7^ Clearly defined values and goals also help make the multiple decisions along the career journey easier. The more decisions one makes, the more likely the next decision will either not get made or will result in a poor choice.^8^–^10^ By limiting viable choices and reducing decision issues, established values smooth the deliberate professional’s journey.^8^–^10^

Journeys also need a destination. Initially, accidental academicians are like Alice during her adventures in Wonderland, knowing they will get “somewhere” if they just travel long enough.^11^ Vague end goals are more difficult and less likely to be achieved than specifically delineated goals.^12^ Combining personal characteristics and micromotives to find a fulfilling end goal enhances goal achievement.^13^ This end goal should be built upon multiple smaller, stepping-stone goals that have two purposes.^14^ In addition to specifically delineated goals, understanding the process required to achieve goals and building goal-reaching processes that reflect and are shaped by their identity in a goal-achieving feedback loop also result in a higher likelihood of goal achievement.^15^–^18^

Understanding stepping-stone goals first helps deliberate professionals to avoid distracting, non-relevant projects, allowing them to travel in the same direction for long distances.^19^–^20^ Attempting to finish all projects, no matter what purpose they serve, results in minimal movement in any one direction.^19^ Stepping-stone goals also permit deliberate professionals to refine their path. These checkpoints help education scholars avoid both hyperbolic discounting^a^ and unintentionally getting too far off track.^21^ These checkpoints serve as a place to reflect upon progress and adjust the endpoint and path based on how beliefs and interests have changed rather than aiming directly for an established goal without consideration of whether that goal remains the best outcome.^22^ To this end, deliberate professionals combine two goal achievement strategies (plan-and-implement^b^ and test-and-learn^c^) to find their individualized path to being an education scholar, strategies used to become a clinician.^23^

### Guidance and How to Travel

Since no universal defined path toward becoming an education scholar exists, deliberate professionals must use travel companions to help plan their route, choose their method of travel, and plan how to choose a direction at any unexpected forks in the road.

A social network guides individualization of one’s path by helping to identify one’s unique qualities, micromotives, strengths and weaknesses.^18^ Four types of people (their tribe^d^, mentors^e^, coaches^f^, sponsors^g^) make up one’s social network. This group provides feedback, suggests opportunities for growth and advancement, and teaches about training and resources required along the journey.^24^–^26^ While some of these relationships occur naturally, others benefit from formalization. Deliberate professionals consciously establish these relationships, determining what each person can and is comfortable providing help with, ensuring they receive the specific guidance they need and support when the journey gets arduous.^24^–^25^ Failure to formalize such relationships results in less guidance and fewer benefits.^56^

In addition to support and guidance, travel companions offer opportunities of varying usefulness.^26^ Without a solid method for choosing which opportunities to accept, the default is often to take the easiest route (ie, saying yes) despite the choice not being beneficial.^27^ This path may hold little to no interest to the education scholar, resulting in procrastination and lack of productivity.^19^ Committing to these opportunities decreases the time available for more interesting and relevant areas, while delaying niche development, something that helps prevent burnout, an increasingly common downside of a medical career.^28^–^29^ Deliberate professionals set themselves up to deal with any forks in the road before they start traveling, reducing decision-making issues and increasing productivity.^9^–^10^

A solid decision tree starts with a foundation of four times to definitely say yes: (1) politically, “no” is not an option (mandated yes); (2) the opportunity definitely interests the traveler; (3) participation in the opportunity directly impacts at least one established goal; and (4) participation results in working with someone who can educate and guide, leading to further success of the journey.

Some opportunities neither fit into these categories nor are an obvious “no.” Setting up priorities, such as how the opportunity might impact the final goal, who is involved, when the opportunity is, or how the traveler would be involved, helps one avoid defaulting to “yes.” Consider also asking and honestly answering questions about the opportunity’s effect on the journey: “What will I have to give up if I say yes?”; “What could I potentially miss out on if I say no?”; “Does this align with who I want to be?”^19^,^30^ The ultimate goal of this prioritization is to make sure travelers are not just “busy,” so travelers are able to both act as well as think and question, allowing germination and growth of ideas.^31^

Discussion of prioritization of opportunities brings up questions of how one should travel on this journey, (ie, how to work productively). A full discussion of productivity techniques is beyond the scope of this commentary. Accidental academicians should reflect upon their personal characteristics to figure out which techniques will work best for them. Deliberate professionals commit to maximizing their productivity. Though physician schedules rarely permit the four-hour blocks that many productive people use, a successful approach to blocking off professional time away from clinical work considers five key issues:^32^

- Consistency: Block off time regularly, preferably at the same time of day.^33^ The more often one works, even if it is just for 10 minutes, the more likely it will become a habit.^34^
- Self-awareness: Schedule this work block during their most productive time of day while employing their best techniques of blocking work within the larger time block (eg, Pomodoro technique^h^, clock-time vs event-time^i^).^35^–^36^
- Boundaries: Both physical and scheduling boundaries help prevent interruptions and increase productivity.
- Just do it: Sit down and start working no matter the lack of motivation or ideas. To do otherwise minimizes output.^37^
- Extra time and deadlines: Understand that everything takes longer than planned and allow for extra time to complete the work involved.^38^ Setting deadlines also helps avoid procrastination.^39^

### Off the Beaten Path

At this point, education scholars must decide which type of trip they will take: traveling straight through with minimal stops or potentially making this a long journey starting with a nearby initial destination with time for detours, viewpoints and rest stops. The first option, without time for anything else until the job is finished, wreaks havoc on work-life integration and makes one’s career less satisfying overall.^40^ Downtime and detours enhance work-life integration and permit discovery and development of niches and passions.^32^

#### Roadblocks/getting lost

Failure, often the most disheartening part of any journey, occurs for everyone. Failure surprises those with a fixed mindset, derailing them from their travels.^41^ With a growth mindset, deliberate professionals do not take failure personally and use failure to their advantage.^41^ They expect failure, understanding it is part of any learning process.^42^ They reframe failure as learning ways that do not work. Finally, deliberate professionals reflect upon their failure and how it affects their career path, modifying their route as needed.^43^

#### Scenic route

Interesting side trips help travelers remain energized for their entire journey. A focus on completing all projects leaves little time for other opportunities. This focus towards the future may result in getting bored with the present and missing beneficial opportunities that do not align perfectly with what was planned.^44^ Deliberate professionals explore intriguing opportunities that pass the decision tree described earlier. Some of the most successful people have had career journeys that involved intentionally entered backward and sideways trips.^45^

#### Rest stops

Deliberate professionals recognize how time outside of work enhances the quality of their work. Prominent historical figures interspersed work with activities like walks, social interactions, and naps, a practice supported by multiple research studies.^32^ K. Anders Ericsson’s deliberate practice needs the support of deliberate rest and sleep for practitioners to achieve expertise.^46^ Deliberate rest (or play) involves detaching from work by changing context, relaxing, and engaging in interesting activities.^47^–^48^ Each traveler’s form of deliberate rest/play is different, but has the same benefits: better performance during work time; renewed energy; and increased creativity.^47^–^50^ Switching off work, especially in the middle of a project, allows the brain to subconsciously consider the problem and arrive at more creative solutions.^32^, ^51^–^52^ Deliberate professionals realize that coming up with solutions while walking, driving, or showering is not a fluke but an opportunity to be cultivated.

## CONCLUSION

During their career journey, deliberate professionals create an individualized map to follow (modifying it as they go), engage guidance in various forms, and understand that time outside of work enhances their experience and productivity. The added intention that differentiates a deliberate professional from an accidental academician seems simple, but each move is deliberate: unhurriedly, carefully, and attentively studied, considered, and measured before it is taken. Practicing reflection regularly is key to understanding the interplay of all parts of the career journey. Both deliberation and reflection take time, experience, and a willingness to be wrong. No matter where education scholars are in their career journey, they can transform from an accidental academician to a deliberate professional and travel a well-planned path that expects and permits agility while enhancing productivity.

## Footnotes

a Having less motivation to do tedious tasks towards a distant reward.

b Define a goal, figure out how to get there, and strictly follow the path.

c Trying different options to determine which one fits.

d A group of people connected to each other by a leader and/or ideals.

e Provide guidance through sharing of knowledge, advice and experience.

f Observes performances and provides feedback to improve performance.

g People of higher rank that can offer opportunities to the education scholar and promote them to others.

h Working in blocks of time (typically 25 minutes) with breaks in between of varying lengths depending on the overall amount of time one has been working.

i Working for either a specified amount of time (clock time) or until a task is finished (event time).
